# Shanghai fever, a fatal enteric illness, in an adult patient with neutropenia caused by treatment-related myelodysplastic syndrome: a case report

**DOI:** 10.1186/s40792-022-01426-5

**Published:** 2022-04-18

**Authors:** Taichi Horino, Tatsunori Miyata, Mitsuhiro Inoue, Kosuke Ono, Asuka Ono, Yoshiyuki Tagayasu, Daichi Nomoto, Takao Mizumoto, Tatsuo Kubota, Nobutomo Miyanari, Hideo Baba

**Affiliations:** 1 Department of Surgery, National Hospital Organization Kumamoto Medical Center, Ninomaru 1-5, Chuo-ku, Kumamoto, 860-008 Japan; 2 Department of Gastroenterological Surgery, Graduate School of Medical Sciences, Kumamoto University, Kumamoto, Japan

**Keywords:** Shanghai fever, *Pseudomonas aeruginosa*, Bowel perforation, Treatment-related MDS

## Abstract

**Background:**

Shanghai fever is a rare community-acquired enteric illness with sepsis caused by *Pseudomonas aeruginosa*. Cases of Shanghai fever in pediatric patients have been reported; however, to the best of our knowledge, there are no reports of adult cases.

**Case presentation:**

A 65-year-old man visited the emergency department with sudden onset of abdominal pain. He was diagnosed as treatment-related myelodysplastic syndrome after treatment of follicular lymphoma. Moderate tenderness in the middle right abdominal quadrants was noted. Computed tomography showed abdominal free air with a small amount of effusion to the surrounding edematous small intestine, and we performed emergency exploration. During operation, we found multiple bowel perforations with patchy necrotic lesions. The patient was admitted to an intensive care unit postoperatively. Blood culture showed *Pseudomonas aeruginosa*. His condition improved; however, on the 8th postoperative day, the abdominal drain tube showed turbid drainage. We performed re-operation and found anastomotic leakage with two new bowel perforations. After the re-operation, the patient showed signs of septic shock and his general condition got worse, and the patient died due to multiple organ failure on the 12th postoperative day.

**Conclusion:**

Shanghai fever may occur in an adult patient with neutropenia.

## Introduction

Shanghai fever is a rare community-acquired enteric illness with sepsis caused by *Pseudomonas aeruginosa*, which leads to serious complications with high mortality [1]. It was first described in 1918 [2]. The most common clinical manifestations are fever (100%), diarrhea (96%), and shock (81%) [1]. Furthermore, necrotizing enteritis with or without bowel perforation, ecthyma gangrenosum, and seizures are reported to be the main complications [3]. Chung et al. reported the following diagnostic criteria of Shanghai fever: (A) community-onset diarrhea with fever, (B) sepsis, and (C) growth of *Pseudomonas aeruginosa* from blood or another sterile body site [1].

Cases of Shanghai fever were reported in children without pre-existing conditions; however, there are no reports of Shanghai fever that occurred in an adult patient with immunodeficiency.

## Case presentation

A 65-year-old man visited the emergency department with sudden onset of abdominal pain that started about 12 h before. He also presented watery diarrhea. He had an 8-year history of follicular lymphoma, and he had undergone various chemotherapy treatments. There had been no sign of recurrence for 8 months owing to response to the chemotherapy. Four months before his current episode, he was diagnosed as treatment-related myelodysplastic syndrome (MDS), and he was prepared for allogenic hematopoietic stem cell transplant. His baseline neutrophil count was 50–200/μL. He was administered oral prednisolone (30 mg/day) for 2 months.

**Fig. 1:**
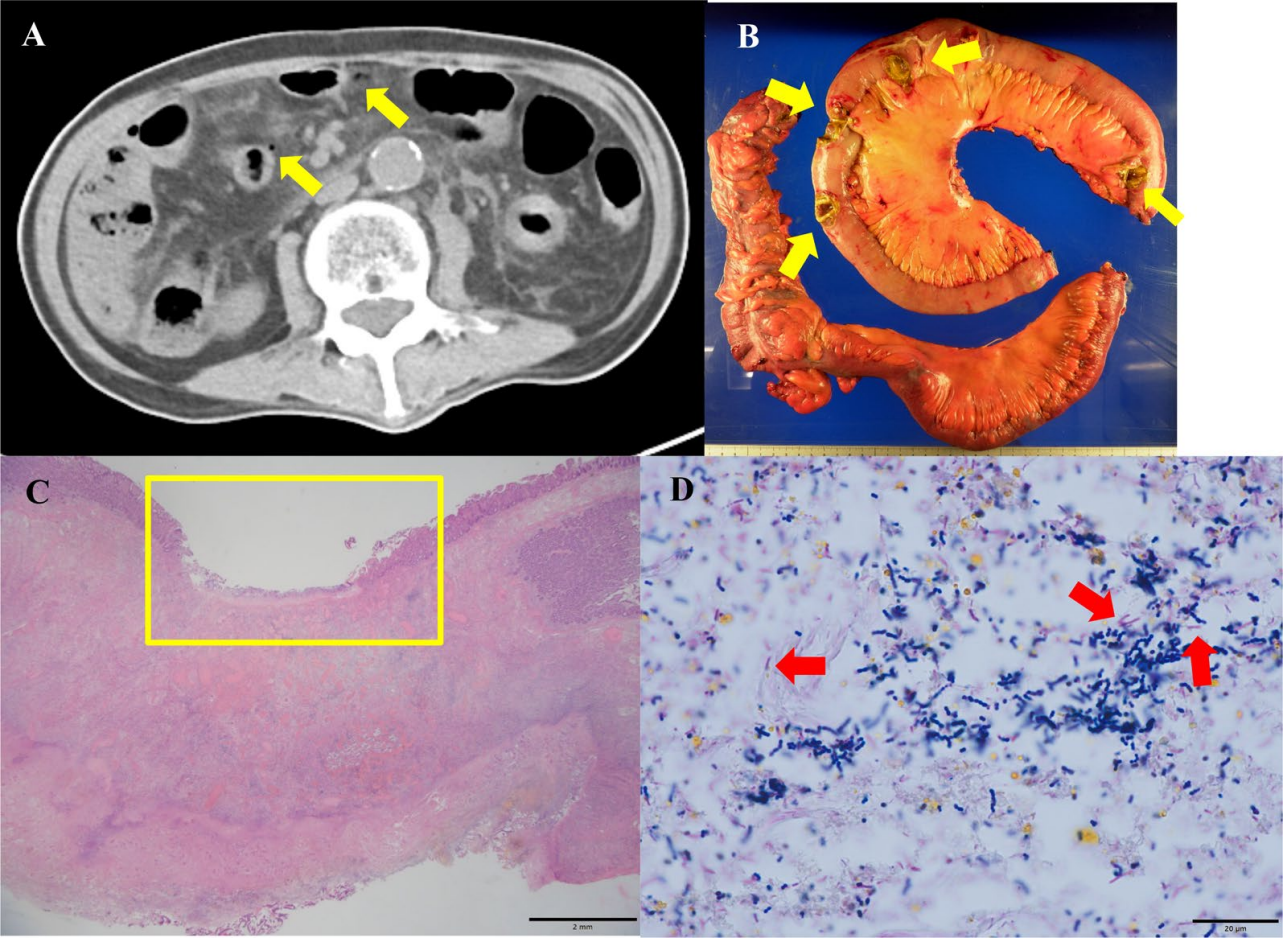
**A** Plain abdominal computed tomography showing free air (yellow arrows) with a small amount of effusion to the surrounding edematous small intestine. **B** Macroscopic image of the resected specimen. Bowel perforations with patchy necrotic lesions (yellow arrows) are shown. **C** Hematoxylin–eosin stain of histopathologic image showing ulcer (yellow enclosing line) of ileum. **D** Gram stain showing proliferation of Gram-negative rods (red arrows) with Gram-positive rods and cocci near the perforated area

On arrival, his vital signs were febrile (body temperature = 38.5 °C) with tachycardia (heart rate = 132 beats per minute). His blood pressure was 108/83 mmHg and respiratory rate was 31 per minute. Upon physical examination, the patient’s abdomen was flat and hard, and moderate tenderness in the middle right abdominal quadrants was noted. Laboratory examinations revealed white blood cell count of 380/μL, neutrophil count of 250/μL, C-reactive protein level of 24.4 mg/dL, and lactate level of 2.6 mmol/L. Plain abdominal computed tomography showed abdominal free air with a small amount of effusion to the surrounding edematous small intestine, but there were no signs of bowel ischemia such as pneumatosis intestinalis or portal venous gas. Based on these findings, we excluded ischemic disease of bowel including non-occlusive mesenteric ischemia or mesenteric arterial thrombosis. The preoperative diagnosis was panperitonitis due to small bowel perforation, and we decided to perform emergency exploratory laparotomy (Fig. 1).

During operation, we found multiple bowel perforations (20-cm, 50-cm, 70-cm, 80-cm, 270-cm, 350-cm, and 360-cm perforations of the jejunum, ileum from the duodenojejunal flexure, ascending colon, and transverse colon near the hepatic flexure) with patchy necrotic lesions. There were no signs of bowel ischemia. We performed small bowel resection and right hemicolectomy including all of the lesions. The postoperative diagnosis was panperitonitis due to multiple bowel perforations.

The patient was admitted to the intensive care unit with respiratory support because he presented fever and hypotension, which suggested septic shock. He also presented watery diarrhea postoperatively. The two sets of blood culture collected on the day of operation showed *Pseudomonas aeruginosa*. Both urine culture and ascites culture were negative for *Pseudomonas aeruginosa*. No other bacterial culture was collected preoperatively. The patient fasted and we started intravenous antibiotics (tazobactam/piperacillin 13.5 g/day) and intravenous prednisolone (30 mg every other day). On the 4th postoperative day, his general condition got better, and the patient was extubated. On the 7th postoperative day, the patient started oral ingestion.

On the 8th postoperative day, the patient complained of right lateral abdominal pain with sudden onset. He was febrile and the abdominal drain tube showed turbid drainage. The diagnosis of postoperative anastomotic leakage was made, and we performed emergency exploration again. During the re-operation, we found anastomotic leakage with two bowel perforations in the remaining ileum and descending colon. We performed small bowel resection and left hemicolectomy with ileostomy and colostomy. After re-operation, the patient showed signs of septic shock and his general condition got worse. On the 12th postoperative day, the patient died due to multiple organ failure.

The pathological examination of the resected intestine showed multiple ulcer and perforation with proliferation of rod-shaped bacilli. Though it suggested the proliferation of *Pseudomonas aeruginosa*, we could not identify the species.

## Discussion

Cases of Shanghai fever were subsequently reported in children without pre-existing conditions, primarily from Asian countries [4–6]. However, to the best of our knowledge, this is the first report of Shanghai fever that occurred in an adult patient with immunodeficiency.

In the present case, the patient presented diarrhea with fever and septic shock with *Pseudomonas aeruginosa* infection, proven by the two sets of blood culture. No other species were proven from blood cultures. Hence our case met the criteria of Shanghai fever [1]. Furthermore, widespread patchy necrotizing intestinal lesions with multiple bowel perforations were found during both first and second operation, which was similar to the reported case of Shanghai fever [1]. Actually, pathological examination of the perforated bowel lesions showed proliferation of Gram-positive and negative rods and Gram-positive cocci near the perforation site. These bacteria are suggested to be both causative agent and indigenous bacteria of intestine. We could not perform autopsy because we respected the intention of his family, so that we could not examine the lesions in further detail. Therefore, we were unable to identify the species of rods under this limitation. We clinically diagnosed the case as Shanghai fever.

Among patients receiving chemotherapy, neutropenia is known to be a major risk factor for *Pseudomonas aeruginosa* infection. The bloodstream, respiratory, and urinary tracts are common infection sites [1]. Persistent lung infection is a known severe condition [7]. In the present case, neutropenia caused by treatment-related MDS may be a risk factor of infection. Furthermore, the patient was administered long-term steroid therapy. Steroid use may cause immunosuppression and is reported to be a risk factor of urinary tract infection caused by *Pseudomonas aeruginosa* [8]. In the present case, prednisolone use may be a risk factor of infection and neutropenia.

There are some diseases with a similar clinical course. Notably, gastrointestinal Behçet’s disease involves the entire small bowel, and especially manifests with round-shaped deep ulcers in the ileocecal region [9, 10]. Moreover, some studies have shown a relationship between MDS and Behçet’s disease [11]; however, in these studies most of the patients had coexistent intestinal Behçet’s disease with ulcers of the ileum without eye lesions [12, 13]. Recently, Oka et al. reported that acquisition of trisomy 8 was associated with Behçet’s disease in MDS [14]. The present patient had no other symptoms that met the international criteria for Behçet’s disease [15], and he was negative for trisomy 8.

Pathological examination showed multiple ulcers and perforation with proliferation of rod-shaped bacilli. The rod was negative for periodic and Schiff stain. Gram staining of the specimen showed Gram-positive and negative rods and Gram-positive cocci near the perforated area. The corresponding clinical manifestations may be present in abdominal tuberculosis, histoplasmosis, sarcoidosis, eosinophilic gastroenteritis, and systemic mastocytosis [3]; however, pathological examinations showed no characteristic signs of such diseases. *Pseudomonas aeruginosa* infection was proven by blood culture; thus, we suspected that the pathogen was the cause.

We performed small bowel resection and right hemicolectomy including all of the lesions. Though both high dose corticosteroid use and septic shock are risk factors of anastomotic failure [16]. In this case, avoiding anastomosis would have resulted in a double stoma with jejunostomy and colostomy, which would have significantly decreased his quality of life. Therefore, we performed intestinal anastomosis in the 1st operation. Indeed, in high-risk cases of anastomotic failure, we always should consider the need for colostomy.

The mortality rate of Shanghai fever is relatively high, as it is associated with multiple organ failure. Halder et al. reported that the mortality rate is 23–89% and that the key treatment of Shanghai fever is general intensive care and antipseudomonal agents [3]. We continued intensive care with antibiotics (tazobactam/piperacillin 13.5 g/day) for 12 days, but the patient succumbed to multiple organ failure.

## Conclusions

Shanghai fever may occur in adult patients with neutropenia. We should consider Shanghai fever for patients with multiple bowel perforation with proven *Pseudomonas aeruginosa* infection, and should start intensive care with antipseudomonal agents.

## Data Availability

All data generated or analyzed during this study are included in this published article.

## Abbreviation

MDS: Myelodysplastic syndrome
